# Torque Analysis of a Triple Acid-Etched Titanium Implant Surface

**DOI:** 10.1155/2015/819879

**Published:** 2015-10-12

**Authors:** Ana Emília Farias Pontes, Cássio Torres de Toledo, Valdir Gouveia Garcia, Fernando Salimon Ribeiro, Celso Eduardo Sakakura

**Affiliations:** Master of Science Program, Educational Foundation of Barretos, UNIFEB, 14783-226 Barretos, SP, Brazil

## Abstract

The present study aimed to evaluate the removal torque of titanium implants treated with triple acid etching. Twenty-one rats were used in this study. For all animals, the tibia was prepared with a 2 mm drill, and a titanium implant (2 × 4 mm) was inserted after treatment using the subtraction method of triple acid etching. The flaps were sutured. Seven animals were killed 14, 28, and 63 days after implant installation, and the load necessary for removing the implant from the bone was evaluated by using a torque meter. The torque values were as follows: 3.3 ± 1.7 Ncm (14 days), 2.2 ± 1.3 Ncm (28 days), and 6.7 ± 1.4 Ncm (63 days). The torque value at the final healing period (63 days) was statistically significantly different from that at other time points tested (ANOVA, *p* = 0.0002). This preliminary study revealed that treatment with triple acid etching can create a promising and efficient surface for the process of osseointegration.

## 1. Introduction

A predictable way to achieve long-term clinical success with dental implants is to ensure the intimate contact between the living bone and the implant, known as osseointegration [1, 2]. More specifically, recent studies have focused on strengthening this interface and accelerating bone formation and healing time, resulting in a rapid recovery of function [3, 4], greater satisfaction of the dentist with the results, and improved quality of life for the patient.

Studies have shown that, compared to a smooth surface, rough topography enhances the osseointegration [5, 6]. To produce a rough surface, various surface modification techniques, such as blasting particles, acid etching, anodizing, adding bioactive molecules, and modification by laser, have been developed and applied, as reported by Piattelli et al. [7].

Among the alternatives for surface conditioning, acid subtraction appears to be an option for production of a rough surface without the presence of residual contaminants [5, 8]. Much of the industry uses this process for the finishing of implants, making it possible to standardize the topography, reduce discrepancies between peaks and valleys on the surface, remove encrusted surface particles, produce microtexturing on a previously treated surface, and eliminate the grooves resulting from the machining process [3, 7].

An innovative surface was produced by triple acid exposure, resulting in coated pores ranging between 1.9 and 2.2 *μ*m. It was proposed that this surface may make the treatment more efficient and accelerate osseointegration because the surface topography influences bone integration on a micrometer level [9].

Thus, the present study aims to evaluate the removal torque of titanium implants treated with triple acid etching.

## 2. Methods

This study included 21 adult male Wistar rats, weighing approximately 350 to 450 g. The animals were kept in propylene cages and fed a standard laboratory diet and water* ad libitum*. The ambient temperature was 25°C, and the ambient relative humidity was 55%. The rats were exposed to 12.5 h of light alternating with 11.5 h of darkness.

The animals were submitted to surgery for implant placement in the proximal metaphysis of the left tibia, as previously described by Boldrini et al. [10]. Anesthesia was administered intramuscularly with a combination of ketamine chlorohydrate at a concentration of 0.04 mL/100 g body weight and 2% xylazine at 0.08 mL/100 g body weight. After peritoneal anesthesia, the animals were shaved, and the surface was cleaned with iodized alcohol.

**Figure 1:**
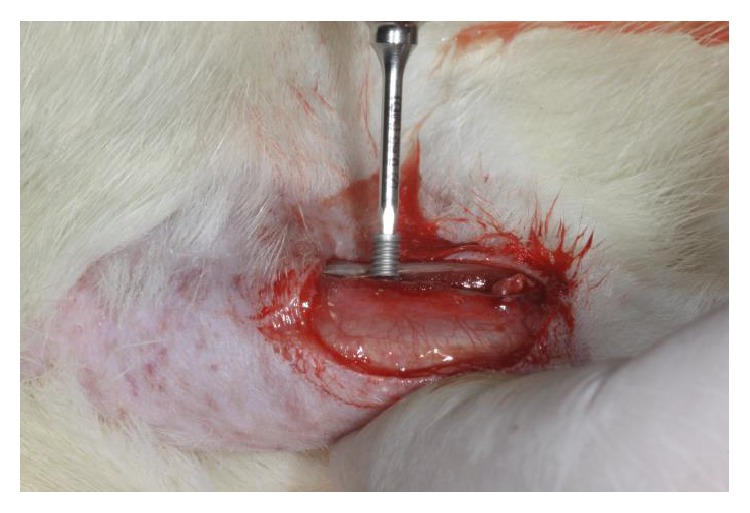
Implant installation.

An incision of 30 mm was made on the internal side of the tibia. After careful dissection, the bone tissue was exposed. Bicortical implant beds were prepared using a 2 mm bur, cooled with saline solution (Figure 1).

In all rats, a titanium microimplant (DentFix, Cambuí, MG, Brazil), with a triple acid-etched surface, 4.0 mm in length and 2.0 mm in diameter, was inserted. After implant placement, the soft tissue was internally sutured with 4.0 polyglactin and externally sutured with 4.0 silk sutures, and the animals received a single intramuscular injection of penicillin with streptomycin and oral administration of paracetamol (15 mg/kg).

Seven animals from each group were killed 14, 28, and 63 days after implant installation with a lethal intraperitoneal injection of 20% chloral hydrate. After that, the tibia was dissected to expose the implant and attach a torque meter (Model ATG24CN-S, Tohnichi, Shanghai, China) with a scale range of 3 to 24 Ncm and divisions of 0.05 Ncm. A wrench was adapted to the implant head to apply torque in the reverse direction of the implant placement until complete rupture of the bone/implant interface was signaled by rotation of the implant. This reading was considered to be the torque necessary to disrupt the osseointegration.

Statistical analysis was performed using specific software (BioEstat 5.0, Sociedade Civil Mamirauá/MCT—CNPq, Belém, Brazil) considering the null hypothesis based on the absence of a difference between different modalities of treatment (alpha = 5%). The Shapiro-Wilk test was used to test the normal distribution of data. An analysis of variance (ANOVA) was followed by the Bonferroni test for comparison of the data related to the removal torque of the implants.

## 3. Results and Discussion

**Figure 2:**
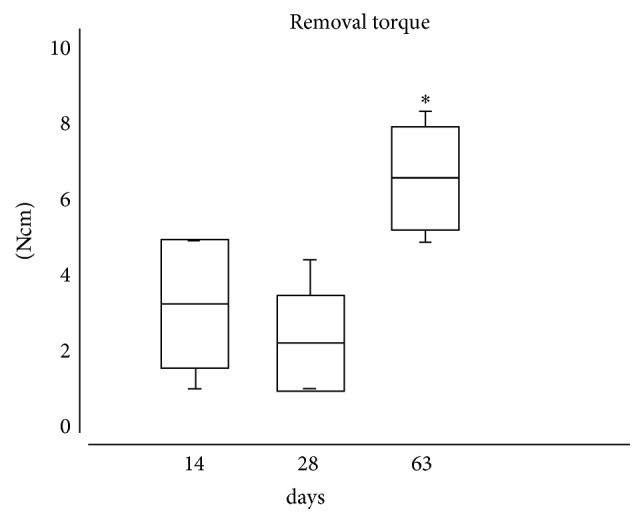
Box-plot representation of the experimental groups (mean, standard deviation, and minimum and maximum values). ^*∗*^Statistically significant difference in comparison to the 14-day and 28-day time points (ANOVA, *p* = 0.0002).

The mean removal torque was 3.3 ± 1.7 Ncm after 14 days of healing, 2.2 ± 1.3 Ncm after 28 days, and 6.7 ± 1.4 Ncm after 63 days (Figure 2). The mean removal torque in final healing period (63 day) was statistically significantly different from that at other time pointes tested (ANOVA, *p* = 0.0002).

The present study aimed to evaluate the removal torque of implants treated with triple acid etching, which were installed in the tibial metaphyses of rats. Data were collected at 14, 28, and 64 days after implantation. There was a small decrease in the value of the removal torque in the samples at 28 days. This finding can be explained by the progress of the bone repair process. After implantation, the primary stability tends to decline during the first month. This is considered a critical stage, in which a high failure rate of the implants is observed. Interestingly, thereafter, the bone-implant interface consolidates, and torque values tend to increase, in a so-called stability secondary phase, until it reaches the full stability [11]. According to Brånemark et al. [12], this phenomenon causes a decrease in the fixation of the implant during the first weeks of healing, and it has been observed in* in vivo* studies to analyze the removal torque.

Specifically regarding the preparation of the surface by acid etching, Hsu et al. [13] argue that this technique reduces the surface concentration of carbon, titanium, and nitrogen, increasing the concentration of oxygen, making it more oxidized than a machined surface. This type of surface conditioning required four times higher torque for removal when compared to the machined surface [5]. Furthermore, this technique allows the creation of a surface with a uniform roughness, without the presence of contaminant particles [8, 14, 15].

Sammons et al. [16] also reported a greater removal force for surfaces submitted to acid etching than that required for machined titanium, plasma spray, and sandblasted surfaces.

Although there is no consensus among researchers regarding the optimal surface roughness and implant design, Eliasa et al. [3] stated that osseointegration can be influenced by the biocompatibility of the material, the surface and shape of the implant, the bone quantity and quality, and even the surgical technique used for implantation. With such a large number of variables involved in the success of osseointegration, additional studies should be conducted to clarify the role and the importance of each in the success of the implant.

This study represents a first step in obtaining a better understanding of the clinical utility of implants prepared with surface acid etching.

## 4. Conclusion

This preliminary study revealed that treatment with triple acid etching can create a promising and efficient surface for the process of osseointegration. However, additional studies are extremely important to determine the surface characteristics and the advantages related to modulation of cellular interaction and to investigate the effects in humans.
